# The Relationship between Cranial Structure, Biomechanical Performance and Ecological Diversity in Varanoid Lizards

**DOI:** 10.1371/journal.pone.0130625

**Published:** 2015-06-24

**Authors:** Matthew R. McCurry, Michael Mahony, Phillip D. Clausen, Michelle R. Quayle, Christopher W. Walmsley, Tim S. Jessop, Stephen Wroe, Heather Richards, Colin R. McHenry

**Affiliations:** 1 Department of Anatomy and Developmental Biology, Monash University, Melbourne, Australia; 2 School of Environmental and Life Science, University of Newcastle, Newcastle, Australia; 3 Geosciences, Museum Victoria, Melbourne, Australia; 4 School of Engineering, University of Newcastle, Newcastle, Australia; 5 Department of Zoology, University of Melbourne, Melbourne, Australia; 6 The Function, Evolution & Anatomy Research Lab, Zoology Division, School of Environmental and Rural Sciences, University of New England, Armidale, Australia; New York Institute of Technology College of Osteopathic Medicine, UNITED STATES

## Abstract

Skull structure is intimately associated with feeding ability in vertebrates, both in terms of specific performance measures and general ecological characteristics. This study quantitatively assessed variation in the shape of the cranium and mandible in varanoid lizards, and its relationship to structural performance (von Mises strain) and interspecific differences in feeding ecology. Geometric morphometric and linear morphometric analyses were used to evaluate morphological differences, and finite element analysis was used to quantify variation in structural performance (strain during simulated biting, shaking and pulling). This data was then integrated with ecological classes compiled from relevant scientific literature on each species in order to establish structure-function relationships. Finite element modelling results showed that variation in cranial morphology resulted in large differences in the magnitudes and locations of strain in biting, shaking and pulling load cases. Gracile species such as *Varanus salvadorii* displayed high strain levels during shaking, especially in the areas between the orbits. All models exhibit less strain during pull back loading compared to shake loading, even though a larger force was applied (pull =30N, shake = 20N). Relationships were identified between the morphology, performance, and ecology. Species that did not feed on hard prey clustered in the gracile region of cranial morphospace and exhibited significantly higher levels of strain during biting (P = 0.0106). Species that fed on large prey clustered in the elongate area of mandible morphospace. This relationship differs from those that have been identified in other taxonomic groups such as crocodiles and mammals. This difference may be due to a combination of the open ‘space-frame’ structure of the varanoid lizard skull, and the ‘pull back’ behaviour that some species use for processing large prey.

## Introduction

The varanoid lizards comprise three modern genera (*Varanus*, *Heloderma* and *Lanthanotus*) and make up important components of various modern predator guilds [1,2]. For example, the monsoonal tropics of Northern Australia contain up to 11 sympatric varanoid species [2] that function as apex predators, mesopredators, insectivores and scavengers [2,3]. Despite having a conserved body plan, varanoids exhibit considerable variation in body size [4,5], limb dimensions [6] and skull structure [7]. Whilst the influence of varanid (referring to the genus *Varanus*) body size on locomotion and ecology has been explored, the consequences and contribution of skull variation for biomechanical and ecological performance have received less attention [5,8,9]. Here we quantify variation in cranial morphology between a number of extant varanoid species and examine the biomechanical and ecological consequences of these differences.

Varanoid lizards were previously considered a monophyletic group [10] however some recent phylogenetic analyses group *Varanus* and *Lanthanotus* together with *Shinisaurus* in Paleoanguimorpha, and find *Heloderma* to be a member of a sister group (the Neoanguimorpha) to these [11]. Within a phylogenetic context, the inclusion of *Heloderma* represents an outgroup to the other specimens included in the study (which are all part of the Paleoanguimorpha). Recent revisions of squamate relationships indicate that the phylogeny may still not be completely resolved [12,13]. Here we use the term varanoid to refer to *Varanus*, *Lanthanotus* and *Heloderma* as a useful ecological or functional grouping, but note that future consensus of phylogenetic analyses may find it to be polyphyletic.

Varanoid lizards exhibit a range of behaviours during capture and processing of prey. Prey is predominantly killed using a bite. If too large to be swallowed whole, prey must be processed into manageable pieces for consumption. To do this varanoid species can shake prey from side to side to tear off chunks [14], or use a sawing pull-back movement where the teeth are drawn back through the item [15,16]. From a biomechanical perspective, a major role of the skull is to transmit the force generated by jaw and postcranial musculature to the prey whilst resisting the loads induced by these behaviours.

Identifying mechanistic links between form and function is challenging because the complex morphology of biological structures, as well as often complex interplay between morphology and performance, makes analytical approaches difficult [17]. Simulation based techniques, such as finite element analysis (FEA), provide a valuable methodology to investigate the effect of complex differences in cranial structure on performance measures [18]. When combined with modern morphometric analyses, these structural models represent a powerful method for predicting the influence of morphology on mechanical performance [19,20,21,22,23]. Palaeontologists and biologists have gained great insights by utilizing FEA to explore how variations in morphology influence structural performance [21,23,24,25,26,27,28,29,30,31,32,33,34,35]. These studies have greatly increased understanding of how (and to what degree) biological form relates to the structural performance (ability to resist loading) of the skull and the influence this has on the animal’s ecology.

The cranial design of varanoid lizards is highly fenestrated compared to mammalian and crocodilian skulls [36]. In engineering design a highly fenestrated structure is termed a “space frame” in contrast to a “shell” construction. In the context of cranial mechanics, it is not clear whether space frame structures exhibit the same form-function relationships as shell constructions. Logically the strength of space frame constructions will be dictated to a high degree by the thickness, position and orientation of the struts, whereas in shell constructions it will be determined by the thickness of the continuous wall that is used to distribute loads. Presumably space-frame skulls are expected to work in a different manner to shell structured skulls with load being transferred through the struts perpendicular to its long axis. The mechanics of shell-type skulls have been investigated a number of times using finite element analysis [24,26,31,32,33,34,37,38,39,40,41,42,43,44]; space-frame skulls have not been examined to the same degree, with only single specimen studies [45,46,47,48,49].

Here we present a comparative cranial FEA of varanoid species, with the aim of quantitatively assessing the relationship between the shape of varanoid lizard crania and mandibles, their structural performance to biologically relevant loading (biting, shaking and pulling forces) and the dietary ecology of the species. Our study includes high resolution models of 13 skulls, making it one of the largest three dimensional finite element analyses of cranial mechanics performed to date.

Beam theory provides a means of calculating the load carrying characteristics of a simple beam structure [50]. Based on beam theory we predicted that:

1. Lower strain levels will occur in taller craniums and mandibles during bite loading.
2. Lower strain levels will occur in wider craniums and mandibles during shake loading.
3. Lower strain levels will occur in taller and wider craniums and mandibles during pull loading.

When comparing performance and ecology it is predicted that:

1. Species that feed on hard prey items will exhibit less strain during bite loading than other species.
2. Species that feed on large prey items will exhibit less strain during shake and pull loading than other species.

## Methods

### Data acquisition and morphometric analysis

**Fig 1:**
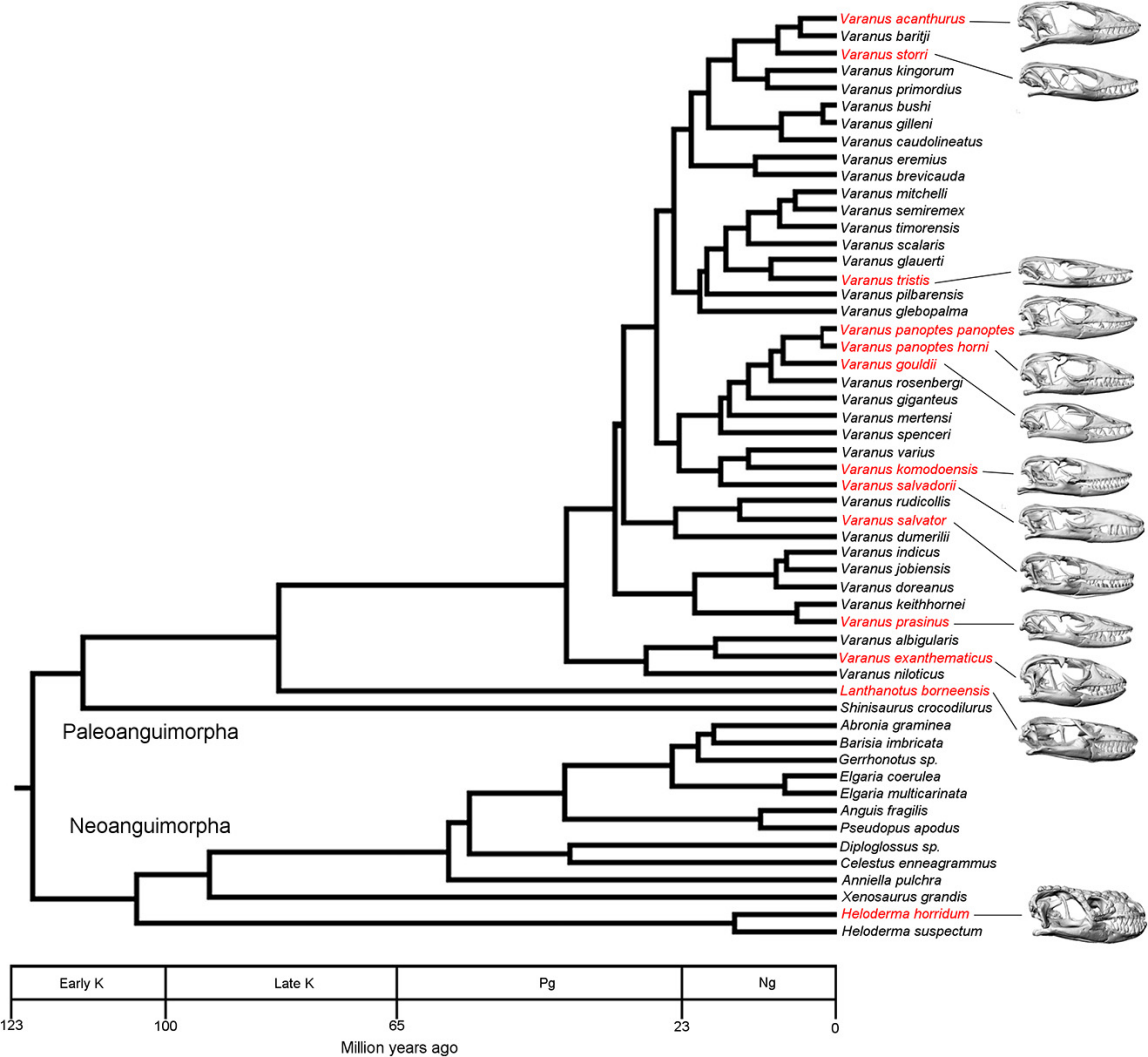
Phylogenetic sampling. Phylogeny of Paleoanguimorpha and Neoanguimorpha showing the taxa used within this study in red text. Labels on timescale: K, Cretaceous; Pg, Paleogene; Ng, Neogene. Adapted from Vidal [11]. Note that the lateral views of the skulls are not to scale.

**Fig 2:**
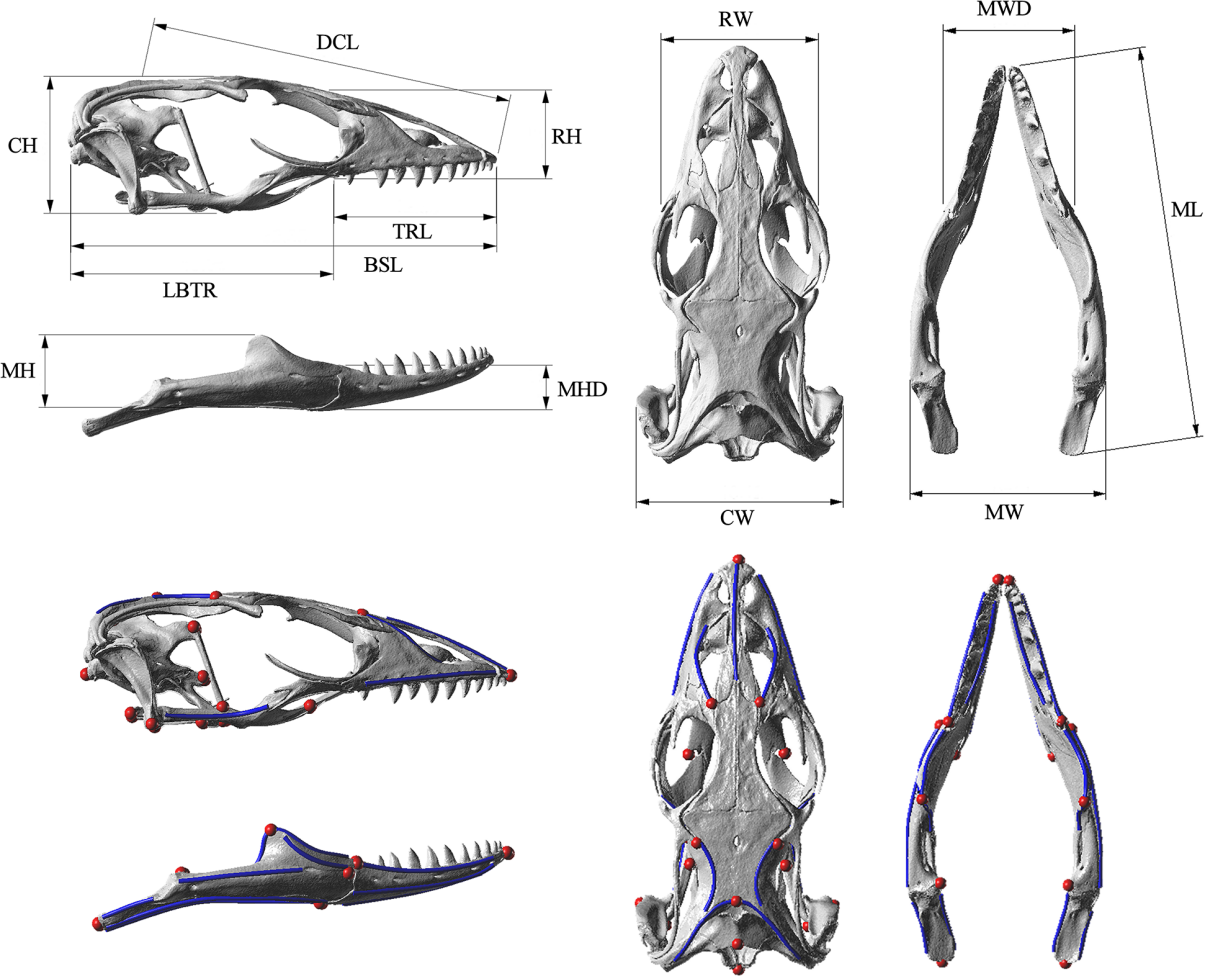
Linear measurements and landmarks used in morphometric analysis. Morphometric information used in this study. Top: linear measurements taken from specimens. Bottom: Points and curves used in geometric morphometric analysis. Point landmarks are shown in red and curves are shown in blue. Abbreviations and definitions for linear measurements can be found in S1 File. The specimen shown is *Varanus acanthurus*: UTA 13015.

Computer tomography (CT) scans of 13 varanoid skulls, representing 12 species, were obtained from online libraries (www.digimorph.org) or through scanning (at either Newcastle Mater Hospital using a Toshiba Aquilion 64 slice CT or at Sydney University using an Xradia MicroXCT-400 micro CT). The specimens were selected from the approximately 79 possible varanoid species to maximise phylogenetic and morphological variation (Fig 1). These specimens are housed in a number of repositories (Table 1). Two of the specimens (*V*. *gouldii* and *V*. *komodoensis*) represent sub-adult specimens. Mimics V13 (MATERIALISE, Belgium) was used to convert scan data into three dimensional surface geometry of the cranium and mandible. Morphometrics were undertaken using both 3D and linear methods. Thirteen linear measurements were taken in the 3D CAD software package Rhino [51] (Fig 2). LANDMARK [52] was used to select the location in 3D space of a series of 24 landmarks and 11 curves on the cranium and 14 landmarks and 22 curves on the mandible for geometric morphometric analysis (Fig 2). These locations were exported as.pts files, manually reformatted, and then imported into MOPHOLOGIKA [53] where Procrustes superimposition and principle component analysis were undertaken (S1 File).

**Table 1:** Specimens used in this study.

Taxon	Specimen number	Repository of original specimen	Source of digital data	Dorsal cranial length (cm)
*Heloderma horridum*	TNHC 64380	Texas Memorial Museum	Digimorph online library	5.7
*Lanthanotus borneensis*	YPM 6057	Yale Peabody Museum of Natural History	Digimorph online library	1.8
*Varanus acanthurus*	UTA 13015	The Amphibian and Reptile Diversity Research Centre at the University of Texas at Arlington	Digimorph online library	2.7
*Varanus exanthematicus*	FMNH 58288	Florida Museum of Natural History	Digimorph online library	4.9
*Varanus gouldii*	AMR 142826	The Australian Museum	Digimorph online library	4.0
*Varanus komodoensis*	AMR 106933	The Australian Museum	Sydney University	11.2
*Varanus panoptes panoptes*	AMR 75084	The Australian Museum	Newcastle Mater Hospital	7.4
*Varanus panoptes horni*	TMM-M-1295	Texas Memorial Museum	Newcastle Mater Hospital CT	5.3
*Varanus prasinus*	AMR 166380	The Australian Museum	Sydney University	3.2
*Varanus salvadorii*	QMJ 14498	Queensland Museum	Newcastle Mater Hospital	11.2
*Varanus salvator*	FMNH 35144	Florida Museum of Natural History	Digimorph online library	11.9
*Varanus storri*	AMR 143912	The Australian Museum	Sydney University	2.2
*Varanus tristis*	AMR 143919	The Australian Museum	Sydney University	3.1

### Finite element analysis

High resolution versions of the geometries used in the morphometric analysis were converted into solid meshes composed of simple tetrahedral elements (tet4s), here termed bricks, using Harpoon (http://www.sharc.co.uk). Each surface.stl file was imported before being meshed firstly using the “wrapping” function and secondly the “all tet” function. The wrapping function allowed for surface and internal geometry to be standardised between models through adjusting the surface cell size and with it the degree of captured geometry. Only major internal geometry such as the mandibular cavity was included. The solid mesh of each cranium consisted of 1 million bricks (±4%). The mandible was then meshed so that the tetrahedral bricks were approximately equal in size to those of the cranium (±1%). These solid models were imported into Strand 7 V2.4.4 (www.strand7.com) for FEA. Isotropic homogeneous material properties (Young’s Modulus = 22.8 GPa, density = 1050 kg/m^3^) were used for bone in all models based on published data of a *Varanus exanthematicus* femur [54]. Homogeneous material properties were chosen over heterogeneous material properties because of: 1) a general lack of data on varanoid bone properties and 2) to better isolate biological shape as a variable within the analysis. A hinge system was implemented to allow the mandible to pivot in relation to the cranium [27] and the gape angle was set to 10 degrees. The jaw hinge was created by extruding beams in the x axis from the lateral side of the bottom of the quadrate. These beams were then subdivided into two equal parts. The end furthest from the skull was released in rotation in the x axis to allow for the jaw to pivot in relation to the cranium. Rigid links were then created to take the place of the half of the beam closest to the skull and to join the freed end to the mandible. A user defined cylindrical co-ordinate system was created around the jaw hinge axis for use in defining the loads (applied forces) and freedoms (used to constrain the model) of each load case.

**Fig 3:**
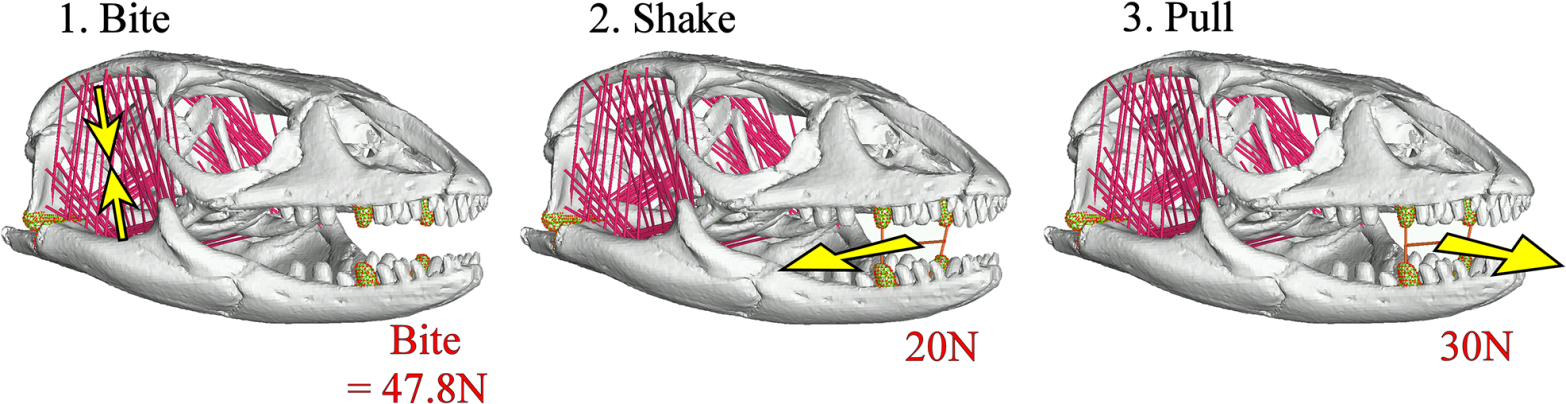
Load cases. The three load cases undertaken in this study: 1. Biting where the models are constructed to bite with the same resultant force of 47.8 N; 2. Shaking where a force of 20N is applied laterally to the middle teeth and 3. Pulling where a force of 30N is applied anteriorly to the middle teeth. Note that within the Shake and Pull load cases muscles do not apply force but brace the skull. The model also shows muscle beams that were used to apply loads in biting (pink), H beams (orange) that were used to apply load to the teeth during shake and pull load cases and surface beams (green) that distribute load across the restrained teeth, jaw hinge surface and occipital condyle. The specimen shown is *Varanus exanthematicus*: FMNH 58288.

Muscles were simulated by attaching 120 truss elements between muscle attachment locations based on the diagrams presented in Holliday [55]. The number of truss elements used to represent each muscle was calculated based on the bone surface areas of the various muscles on *Varanus panoptes horni* (the species of median volume). Muscle elements were defined as either being part of the temporal or pterygoid muscle groups which resulted in 56 temporal truss elements and 64 pterygoid truss elements. Each truss element was assigned a diameter of 1.944423 cm and applied with a tensile force based on the cross sectional area of the two major muscle groups outlined in Walmsley et al.[21] and McHenry [56]. The numbers of beams used to represent each muscle were selected based on the relative surface area of the muscle attachment areas in *Varanus panoptes panoptes*. Table 2 displays which muscles were classified into each of these groups as well as the number of beams used to represent the muscle. Muscle elements were evenly distributed over the attachment areas. The same force was applied to each truss within its respective muscle group (temporal or pterygoid). Data on the forces calculated for the muscles can be found in S1 Table. In whole, this method of model construction assumes that the muscle attachment sites of the various species are identical whilst including differences in the relative size of the two muscle groups. Load cases were used to simulate the forces generated by relevant feeding behaviours. Each model was scaled to the same volume as *V*. *panoptes horni*, following the methods used in Walmsley et al.[21,57]; applied forces were not scaled. The three load cases simulated were:

1. **Bite loading,** where pretension was applied through muscle beams. The occipital condyle was restrained in all 6 degrees of freedom and the four largest teeth in the middle of the tooth row were restrained in circumferential translation centred on the jaw hinge axis. The right upper and lower teeth were also restrained in medial-lateral translation. The magnitude of force applied through each muscle beam (constructed as a “truss” element in Strand7) was standardised as to generate the same node reaction forces at the teeth (i.e. the same resultant bite force) of 47.8 N (Predicted bite force for the *Varanus panoptes horni* model)(Fig 3). The muscle beam forces applied to each model to give that standardised resultant bite force are shown in S1 Table.
2. **Shake loading**, where a force of 20N was applied in a lateral direction using a system of beams connecting the four middle teeth. Muscle beams were set with a Young’s Modulus of 15 MPa with no pretension applied to simulate the muscles bracing the skull [21,56]. The occipital condyle was restrained in all 6 degrees of freedom and the centre of the beams connecting the middle teeth was restrained in an arc around the jaw hinge axis (Fig 3).
3. **Pull back loading,** where a force of 30N was applied in an anterior direction using a system of beams connecting the four middle teeth. Muscle beams were again set with a Young’s Modulus of 15 with no pretension applied to simulate the muscles bracing the skull. The occipital condyle was restrained in all 6 degrees of freedom and the centre of the beams connecting the middle teeth was restrained in an arc around the jaw hinge axis (Fig 3).

**Table 2:** Functional classification of jaw muscles into two muscle groups (temporalis and pterygoid).

Individual muscle	Abbreviation	Muscle group	Number of elements
m. pterygoideus	mPT	Pterygoid	34
m. levator pterygoideus	mLPt	Pterygoid	4
m. protractor pterygoideus	mPPt	Pterygoid	26
m. adductor mandibulae posterior	mAMP	Temporalis	6
m. adductor mandibulae externus superficialis	mAMES	Temporalis	16
m. adductor mandibulae externus medialis	mAMEM	Temporalis	10
m. pseudotemporalis superficialis	mPSTs	Temporalis	16
m. pseudotemporalis profundus	mPSTp	Temporalis	2
m. adductor mandibulae externus profundus	mAMEP	Temporalis	6

A series of beams were also used to reinforce areas where artificial (high) strain was expected. These areas include the restrained teeth, the occipital condyle (also a site of restraint), the jaw hinge surfaces and small areas around the site of each muscle beam attachment.

### Ecological classifications

Data on the diet of species was compiled from relevant scientific literature [58,59,60,61,62,63,64,65,66,67,68,69,70,71,72]. Because of the nature of much of the published data, we were only able to draw broad categories based on the presence or absence of prey items with certain characteristics (Tables 3 and 4). The two categories were: 1) whether the diet of the species included prey items considered as hard, or harder, than a bird egg (e.g. crabs) and 2) whether the diet of the species included prey items larger than could be swallowed whole (e.g. large mammals such as wallabies).

**Table 3:** Ecological classifications.

Species	Hard Prey	Large Prey
*Heloderma horridum*	Y	N
*Lanthanotus bornensis*	Y	N
*Varanus acanthurus*	Y	N
*Varanus exanthematicus*	Y	N
*Varanus gouldii*	N	Y
*Varanus komodoensis*	Y	Y
*Varanus panoptes horni*	Y	Y
*Varanus panoptes panoptes*	Y	Y
*Varanus prasinus*	N	Y
*Varanus salvadorii*	Y	Y
*Varanus salvator*	Y	Y
*Varanus storri*	N	N
*Varanus tristis*	N	N

**Table 4:** Diet of the study species.

Food item	H.hor	L.bor	V.exa	V.slt	V.pra	V.sdi	V.kom	V.gou	V.pan	V.stor	V.aca	V.tri
worms		1										
other insects	1		1	1	1	1	1	1	1	1	1	1
beetles	1		1		1		1	1	1		1	1
orthopterans			1		1			1	1	1	1	1
centipedes			1						1		1	
spiders					1			1	1	1	1	
scorpions			1									
snails			3								3	
mussels									3			
crabs		3		3								
fish		1		2								
small lizards			1			1		1	1	1	1	1
large lizards						1		1	1			
snakes									1			
lizard eggs	1			1				1	1	1		1
amphibians			1	1					1			
birds	1		1	1		1						1
bird eggs	3		3	3		3	3		3			
small mammals				1	2		1		1			
large mammals						2	2					
carrion				2			2	2	2			

1 = normal prey item

2 = food items too large to be eaten whole

3 = hard prey items. Data was compiled from relevant scientific literature. Note that dietary record for several species were incomplete.

### Statistical analysis

**Fig 4:**
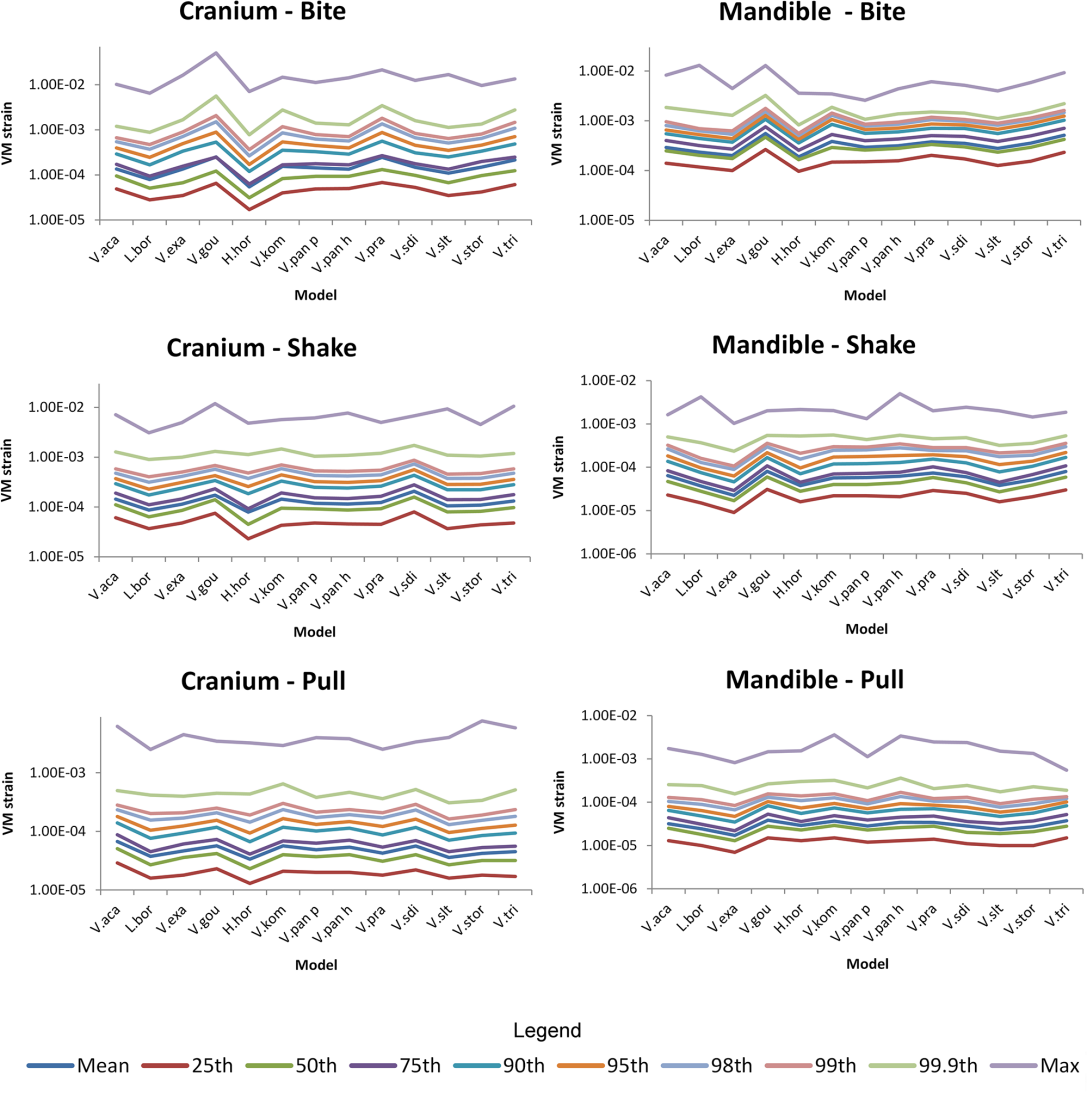
Comparison of possible summary values used as performance measures. Comparison of the pattern between models using various summary values. These were generated from von Mises strain values for each brick within the models. Note that all values, apart from the maximum, 99^th^ and 99.9^th^ values, depict a similar pattern in the results.

The resulting strains for each brick, in either the cranium or the mandible, during each load case were exported to and analysed in R [73]. R was then used to identify the 95% von Mises (VM) strain values of each case following the methods of Walmsley et al. [21,73]; this 95% von Mises strain constitutes the largest elemental (individual brick) value of strain in the model if the highest 5% of all elemental values are removed. The R code has been uploaded as a supplementary file (S2 File) as well as instructions on its use (S3 File). Examination of resulting strain values using mean, 25%, 50%, 75%, 90%, 95% and 98 values all showed similar patterns in results between models. Patterns between models using 99, 99.9 and maximum values did however vary slightly to the others indicating that localised peaks may be affecting these values (Fig 4). The 95% value presumably represents a way to compare the performance of the models whilst ignoring peak loading artefacts.

Tests for phylogenetic signal in the morphometric results (using the phylogeny presented in Vidal [11]) were undertaken using a permutation test with 10000 randomisation rounds in MorphoJ. Partial least squares analysis (PLS) was undertaken in JMP V11 to test the relationship between morphology and the strain levels resulting from FEA load cases. Only one latent variable was included in each comparison because the addition of extra variables did not greatly improve the percentage of variation explained. Variable importance values and percentage variation values were also calculated in JMP. In the case of linear variables, the analysis was run between each single performance measure (VM strain during biting, shaking and pulling) and all morphological variables of the mandible or cranium, the percentage variation explained is only presented for the best correlated morphological variable. We also tested for intraspecific allometry using the same PLS method, comparing centroid size and PC1 and PC2 of the cranium and mandible. Multivariate analysis of variance (MANOVA), with a repeated measures response specification, was undertaken in JMP V 11 to test the relationship between ecological variables and performance. Univariate tests (ANOVA) were then used to test each individual combination of variable after the initial analysis.

## Results

### Ecological classification

All species are opportunistic predators, feeding on a range of vertebrate and invertebrate prey including small lizards, small and large mammals, bird and lizard eggs, amphibians, worms, beetles, centipedes, spiders, crabs, fish and birds. However, noticeable variation in diet is also reported in the published literature [58,59,60,61,62,63,64,65,66,67,68,69,70,71,72]. This variation appears to reflect both habitat and capabilities in prey capture (Tables 3 and 4). No species solely feed on invertebrates, however *V*. *acanthurus* and *V*. *storri* were found to prey on insects along with small prey such as lizards. Other notable specialisation included *H*. *horridum*, which preys primarily on eggs and juvenile birds, and *V*. *komodoensis* and *V*. *salvadorii*, the only species within this analysis to feed on large mammals (Tables 3 and 4).

#### Morphometrics

**Fig 5:**
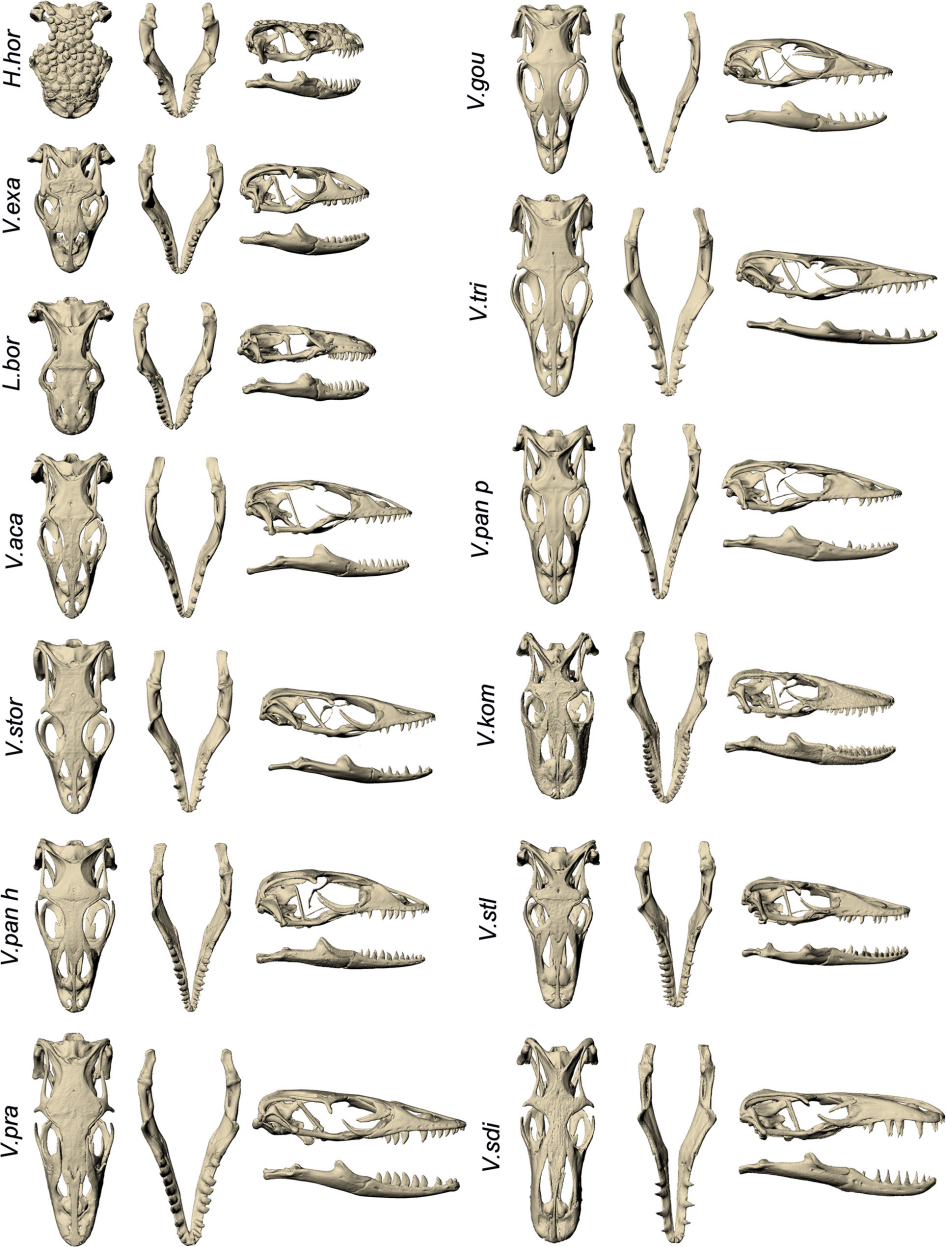
Visual comparison of cranial and mandibular morphology between specimens. The morphology of each specimen is shown in dorsal and lateral view for both the cranium and mandible. Species are ordered by cranial PC1 values. Abbreviations: H.hor, *Heloderma horridum;* L.bor, *Lanthanotus borneensis;* V.aca, *Varanus acanthurus;* V.exa, *Varanus exanthematicus;* V.gou, *Varanus gouldii;* V.kom, *Varanus komodoensis;* V.pan h, *Varanus panoptes horni;* V.pan p, *Varanus panoptes panoptes;* V.pra, *Varanus prasinus;* V.sdi, *Varanus salvadorii;* V.slt, *Varanus salvator;* V.stor, *Varanus storri;* V.tri, *Varanus tristis*.

**Fig 6:**
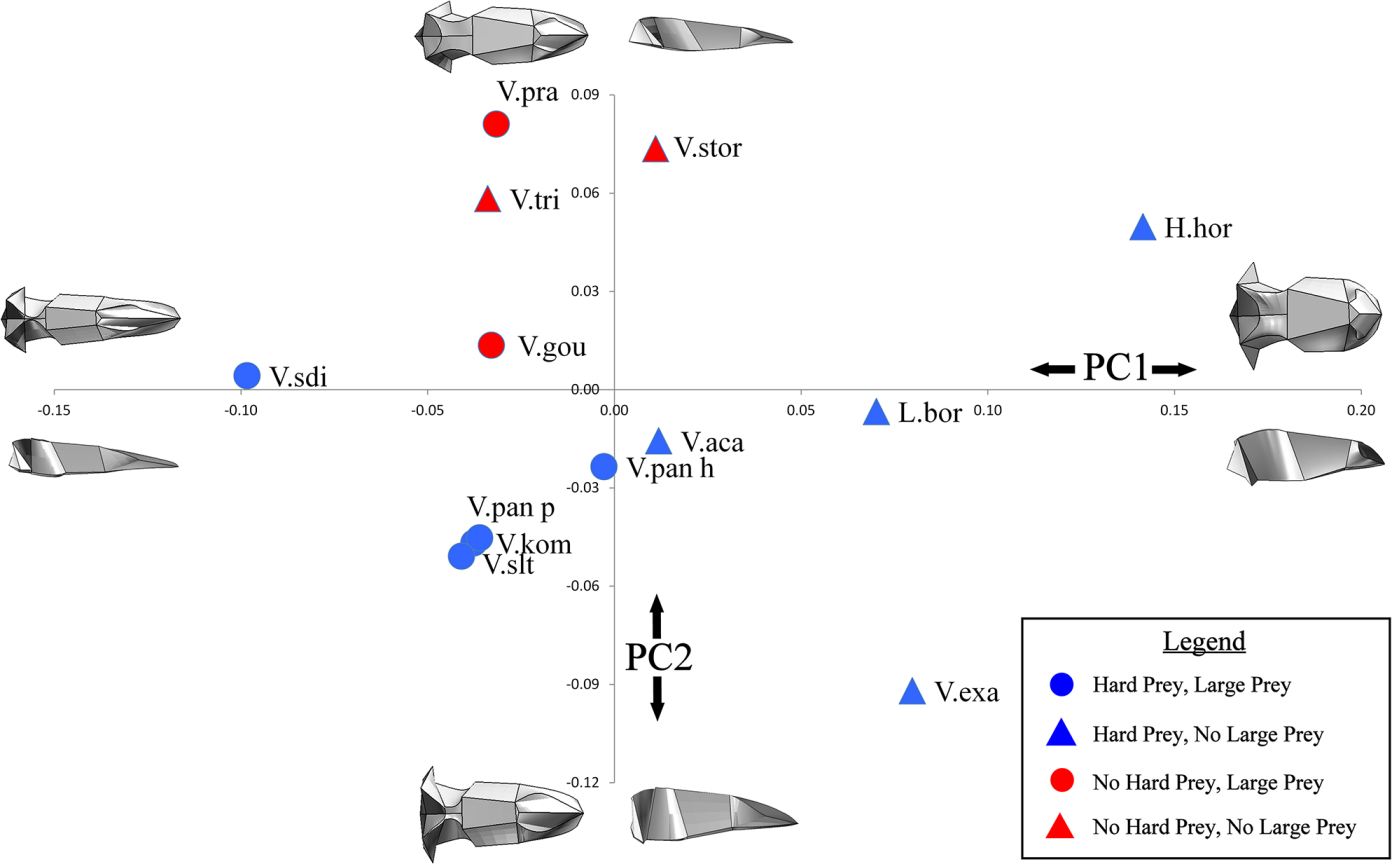
Cranial morphospace. Principal component plot of cranial morphospace. The diagrams at the end of each axis represent the theoretical geometry of the cranium in dorsal and lateral view. Each marker depicts the location of that specimen in morphospace as well as whether the animal was classed as feeding on hard prey (blue = yes, red = no) and whether the animal was classed as feeding on comparatively large prey (circle = yes, triangle = no). Abbreviations: H.hor, *Heloderma horridum;* L.bor, *Lanthanotus borneensis;* V.aca, *Varanus acanthurus;* V.exa, *Varanus exanthematicus;* V.gou, *Varanus gouldii;* V.kom, *Varanus komodoensis;* V.pan h, *Varanus panoptes horni;* V.pan p, *Varanus panoptes panoptes;* V.pra, *Varanus prasinus;* V.sdi, *Varanus salvadorii;* V.slt, *Varanus salvator;* V.stor, *Varanus storri;* V.tri, *Varanus tristis*.

**Fig 7:**
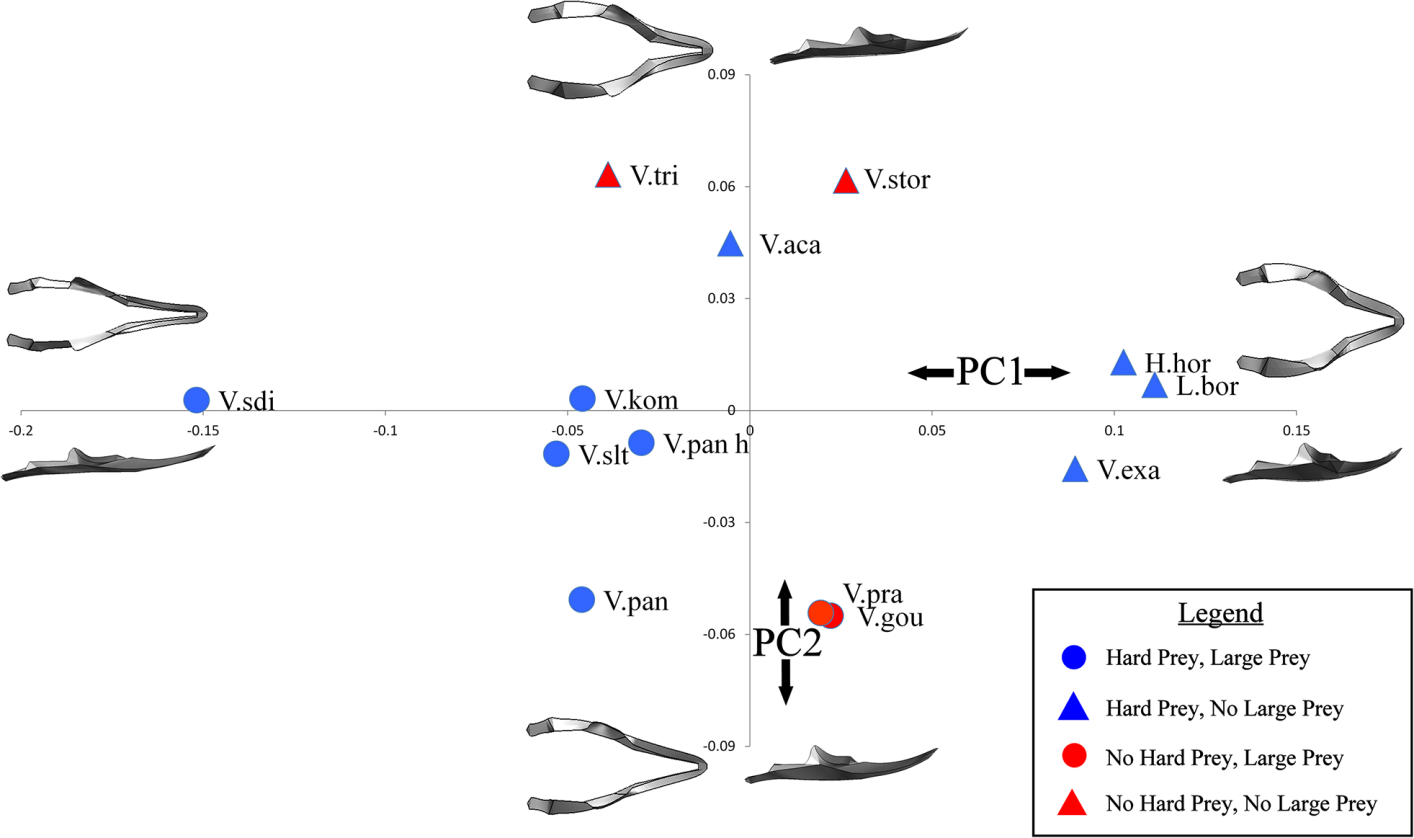
Mandibular morphospace. Principal component plot of mandibular morphospace. The diagrams at the end of each axis represent the theoretical geometry of the mandible in dorsal and lateral view. Each marker depicts the location of that specimen in morphospace as well as whether the animal was classed as feeding on hard prey (blue = yes, red = no) and whether the animal was classed as feeding on comparatively large prey (circle = yes, triangle = no). Abbreviations: H.hor, *Heloderma horridum;* L.bor, *Lanthanotus borneensis;* V.aca, *Varanus acanthurus;* V.exa, *Varanus exanthematicus;* V.gou, *Varanus gouldii;* V.kom, *Varanus komodoensis;* V.pan h, *Varanus panoptes horni;* V.pan p, *Varanus panoptes panoptes;* V.pra, *Varanus prasinus;* V.sdi, *Varanus salvadorii;* V.slt, *Varanus salvator;* V.stor, *Varanus storri;* V.tri, *Varanus tristis*.

Qualitatively, species of varanoid lizards differ substantially in cranial and mandibular morphology (Fig 5). Linear measurements taken on volume scaled models are shown in Table 5. Geometric morphometric analysis provided a more comprehensive picture of morphological variation. Within the cranium, Principal Component (PC) 1 and PC2 together only explained 58.26% of the differences in shape. PC1 was composed of differences in the height, width, length and curvature of the rostrum as well as the width of the skull roof. PC2 was represented by the height of the back of the skull and the area of the skull roof (Fig 6). Within the mandible, PC1 and PC2 together explained 65.07% of the variation in shape. PC1 was composed of differences in the overall width and height of the mandible as well as the position and height of the coronoid process. PC2 represented changes in the height of the coronoid process and comparative length of the tooth row (Fig 7).

**Table 5:** Linear measurements (cm) from volume scaled models.

Species	BSL	DCL	CH	CW	RH	RW	TRL	IOD	ML	MH	MW	MHD	MWD
*Heloderma horridum*	4.11	3.97	3.77	3.06	2.69	3.57	3.11	3.17	4.11	2.58	3.73	2.38	3.20
*Lanthanotus bornensis*	4.30	4.02	3.83	3.03	2.47	3.49	3.27	2.77	4.22	2.55	3.78	2.37	3.41
*Varanus acanthurus*	4.53	4.37	3.83	3.40	2.90	3.54	3.54	2.18	4.50	2.72	3.80	2.28	3.41
*Varanus exanthematicus*	4.20	4.02	3.80	3.19	2.90	3.46	3.37	2.27	4.22	2.73	3.78	2.11	3.38
*Varanus gouldii*	4.55	4.40	3.81	3.32	2.93	3.51	3.72	2.14	4.51	2.72	3.91	2.24	3.51
*Varanus komodoensis*	4.39	4.26	3.65	3.17	2.79	3.55	3.62	2.37	4.48	2.57	3.71	2.33	3.23
*Varanus panoptes horni*	4.45	4.30	3.68	3.37	2.94	3.45	3.61	2.13	4.45	2.66	3.68	2.10	3.30
*Varanus panoptes panoptes*	4.50	4.35	3.59	3.31	2.91	3.44	3.73	2.25	4.51	2.76	3.75	2.35	3.32
*Varanus prasinus*	4.52	4.35	3.53	3.17	2.81	3.41	3.73	2.37	4.45	2.46	3.74	2.08	3.40
*Varanus salvadorii*	4.55	4.39	3.54	3.15	2.75	3.30	3.78	2.10	4.52	2.46	3.59	2.22	3.01
*Varanus salvator*	4.38	4.23	3.71	3.15	2.72	3.33	3.60	2.31	4.41	2.64	3.69	2.41	3.24
*Varanus storri*	4.50	4.34	3.71	3.11	2.79	3.47	3.49	2.12	4.45	2.54	3.76	2.11	3.28
*Varanus tristis*	4.63	4.46	3.73	3.12	2.79	3.50	3.73	2.11	4.59	2.26	3.84	1.89	3.20

Both phylogenetic and functional patterns are present within the results; a permutation test using a time calibrated phylogeny produced a P value of 0.0402 in the cranium and a P value of 0.0018 in the mandible indicating that the dataset contains significant phylogenetic influence [74]. *Heloderma horridum*, *Lanthanotus borneensis* and *Varanus exanthematicu*s clustered in each of the PC plots, the Australian dwarf monitors (subgenus *Odatria*; included here are *V*. *acanthurus*, *V*. *storri*, and *V*. *tristis*) also clustered, occupying the top of the mandible morphospace. The test for interspecific allometry using PLS identified one pair of singular warp vectors that were poorly correlated (Total variation explained for Y effects = 13.467), indicating that allometric trends in shape are small. The species that fed on prey larger than can be swallowed whole clustered in the centre left of the cranial morphospace and in the bottom left of the mandible morphospace. This morphospace is associated with thin and shallow mandibles and thin, long crania with comparatively long tooth rows. The species that feed on large prey items occupied a larger area of morphospace in the mandible analysis than in the cranial analysis (Figs 6 and 7).

### Finite element analysis

**Fig 8:**
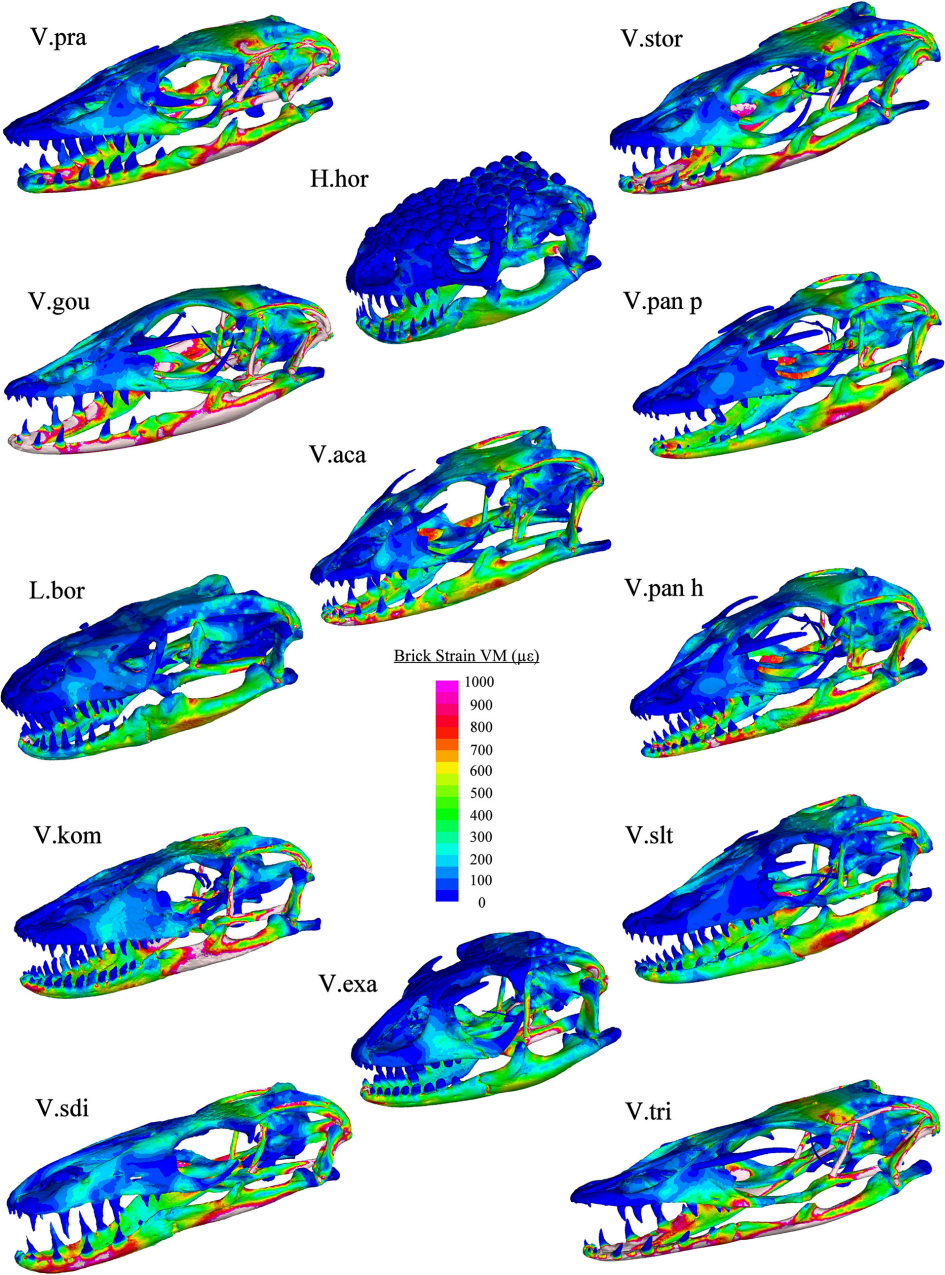
VM Strain plots: Bite loading. Cool colours (blues) represent areas of low stain, hot colours (reds) represent areas of high strain and white areas represent areas where the upper strain threshold (1000 μԐ) was exceeded. Load was applied through muscle beams at a magnitude that standardised bite force at the teeth to 47.8 N (see S1 Table for muscle forces used for each model). Abbreviations: H.hor, *Heloderma horridum;* L.bor, *Lanthanotus borneensis;* V.aca, *Varanus acanthurus;* V.exa, *Varanus exanthematicus;* V.gou, *Varanus gouldii;* V.kom, *Varanus komodoensis;* V.pan h, *Varanus panoptes horni;* V.pan p, *Varanus panoptes panoptes;* V.pra, *Varanus prasinus;* V.sdi, *Varanus salvadorii;* V.slt, *Varanus salvator;* V.stor, *Varanus storri;* V.tri, *Varanus tristis*.

**Fig 9:**
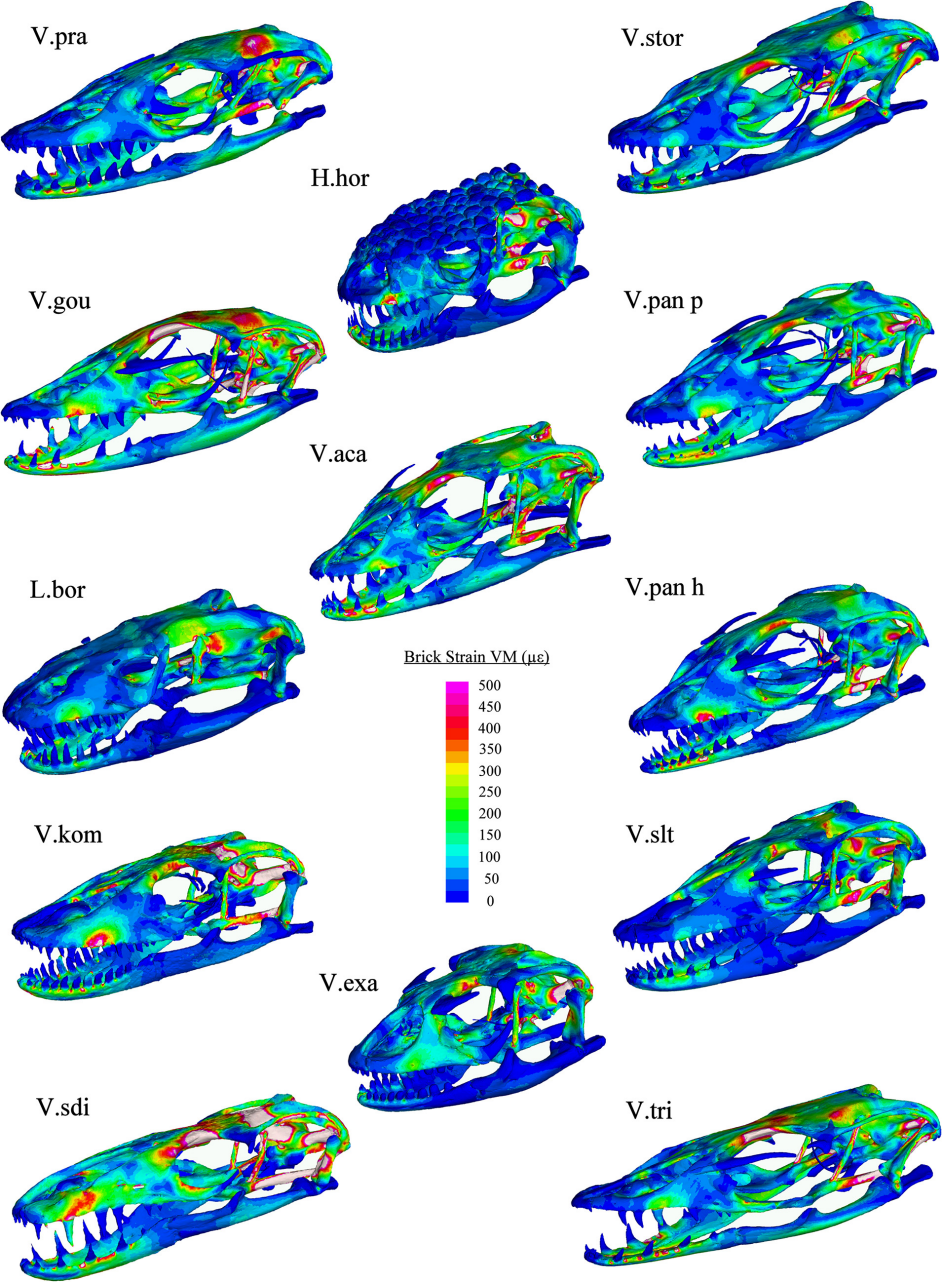
VM Strain plots: Shake loading. Cool colours (blues) represent areas of low stain, hot colours (reds) represent areas of high strain and white areas represent areas where the upper strain threshold (500 μԐ) was exceeded. An arbitrary load of 20N was applied laterally through H beams connecting the four middle teeth. Abbreviations: H.hor, *Heloderma horridum;* L.bor, *Lanthanotus borneensis;* V.aca, *Varanus acanthurus;* V.exa, *Varanus exanthematicus;* V.gou, *Varanus gouldii;* V.kom, *Varanus komodoensis;* V.pan h, *Varanus panoptes horni;* V.pan p, *Varanus panoptes panoptes;* V.pra, *Varanus prasinus;* V.sdi, *Varanus salvadorii;* V.slt, *Varanus salvator;* V.stor, *Varanus storri;* V.tri, *Varanus tristis*.

**Fig 10:**
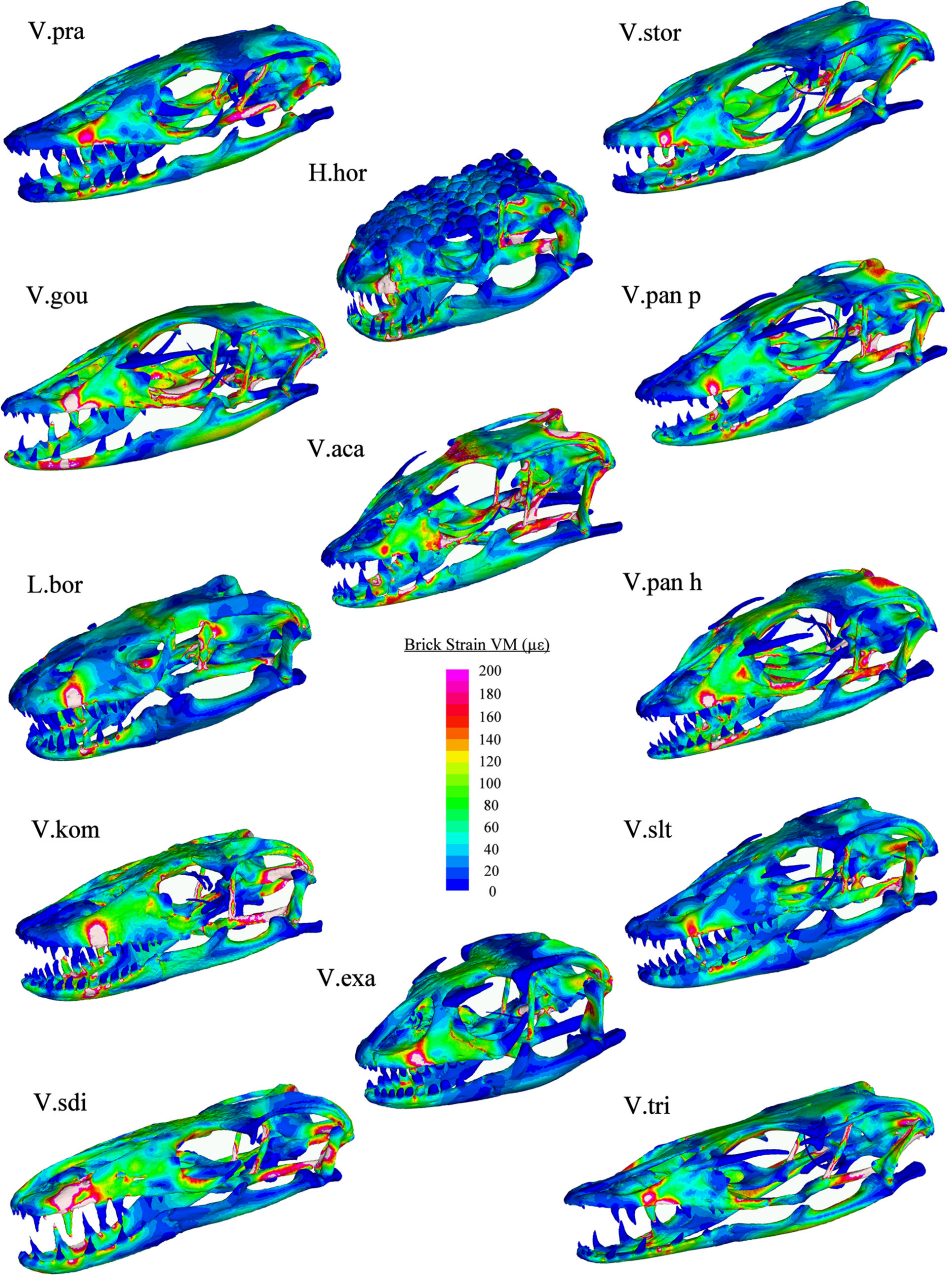
VM Strain plots: Pull loading. Cool colours (blues) represent areas of low stain, hot colours (reds) represent areas of high strain and white areas represent areas where the upper strain threshold (200 μԐ) was exceeded. An arbitrary load of 30N was applied anteriorly through H beams connecting the four middle teeth. Abbreviations: H.hor, *Heloderma horridum;* L.bor, *Lanthanotus borneensis;* V.aca, *Varanus acanthurus;* V.exa, *Varanus exanthematicus;* V.gou, *Varanus gouldii;* V.kom, *Varanus komodoensis;* V.pan h, *Varanus panoptes horni;* V.pan p, *Varanus panoptes panoptes;* V.pra, *Varanus prasinus;* V.sdi, *Varanus salvadorii;* V.slt, *Varanus salvator;* V.stor, *Varanus storri;* V.tri, *Varanus tristis*.

**Fig 11:**
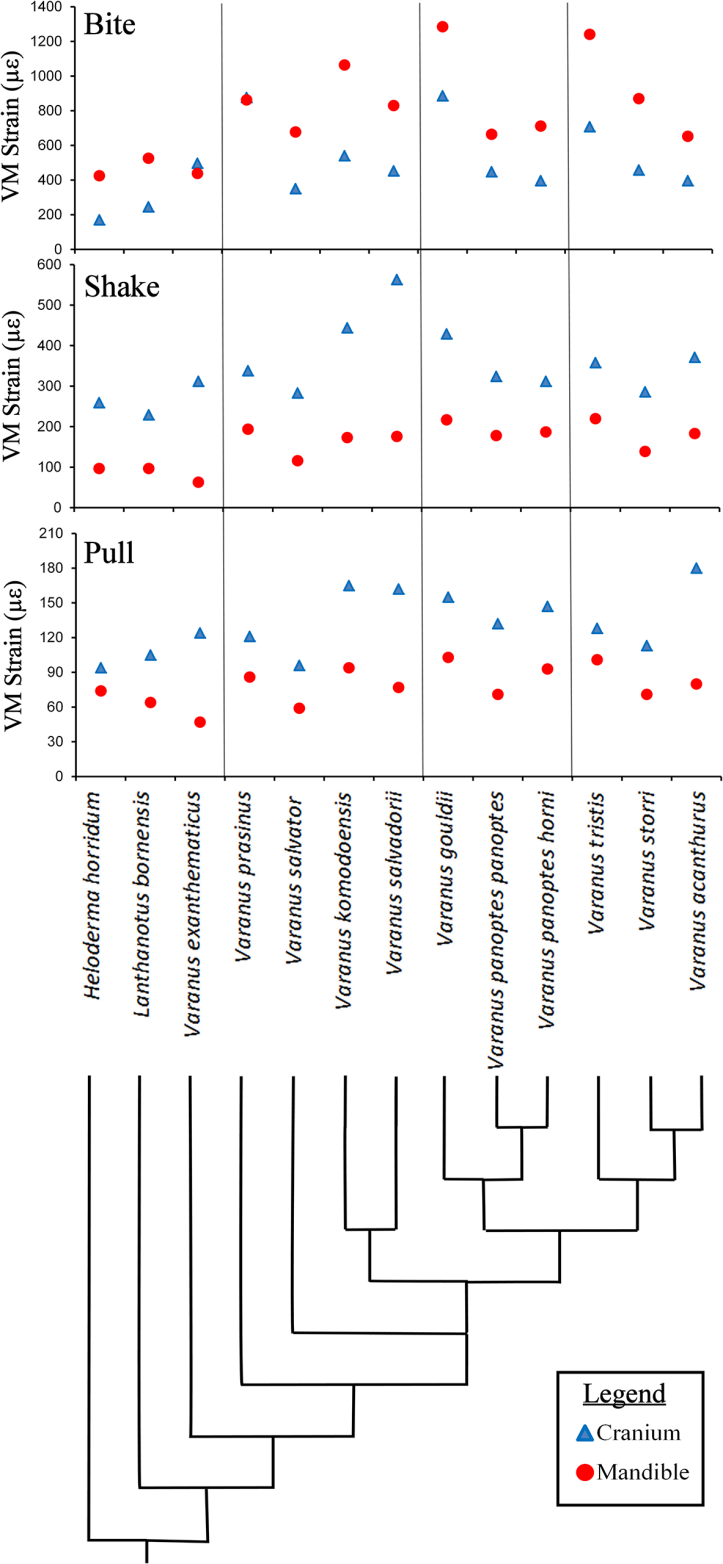
Magnitudes of strain. Comparative performance (95% von Mises strain levels) of the cranium (blue) and mandible (red) models in bite, shake and pull loading. The species are in phylogenetic order with a simplified phylogenetic tree based on Vidal [11] showing the relative position of each species.

Finite element modelling results showed that variation in cranial morphology resulted in large differences in the magnitudes and locations of strain in each load case (Figs 8–11). During bite loading, strain occurred in high levels within the pterygoid, epipterygoid, sides and front of the rostrum as well as in the mandible anterior to the jaw hinge axis (Fig 8). Low magnitudes of strain were observed in the crania of *H*. *horridum*, *L*. *borneensis and V*. *salvator* and the mandibles of *H*. *horridum*, *L*. *borneensis*, and *V*. *exanthematicus* in this load case.

There was considerably less strain associated with the mandible in shake loading compared to bite loading (Fig 11). Patterns of strain are similar in each model during shaking with high levels in the anterior of the mandible and across parts of the cranium, specifically in-between the orbits, the posterior part of the pterygoid bone, the epiterygoid, and the opisthotic and in localised areas close to the teeth at which forces were applied (Fig 9). The magnitude of strain within these areas varied considerably between species (Figs 8, 9 and 10). Gracile species such as *V*. *salvadorii* displayed high strain levels during shaking, especially in the areas between the orbits. *V*. *salvadorii* displayed strain values substantially higher than the other models. The *V*. *salvadorii*, *V*. *komodoensis* and *V*. *gouldii* models exhibited high VM strain values within the cranium. Considerably less variation in strain was observed in mandibles as opposed to the crania during this load case (Fig 11).

The areas of high strain during pull back loading include the pterygoid, epipterygoid and opisthotic bones as well as the sides of the rostrum and areas surrounding the teeth where forces were applied (Fig 10). All models exhibit less strain during pull back loading compared to shake loading, even though a larger force was applied (pull = 30N, shake = 20N). During pull back load cases strain values varied between each model. Within the cranium the highest observed strain values were in *V*. *acanthurus* followed by *V*. *komodoensis*, *V*. *salvadorii* and *V*. *gouldii*. Within the mandible strain magnitudes were high in the *V*.*gouldii* and *V*.*tristis* models (Fig 10). Within intrinsic (bite) loading strain was higher in the mandible than the cranium. In contrast extrinsic (shake and pull) load cases resulted in higher levels of strain in the cranium than the mandible. Overall the results show that each species varies considerably in terms of its ability to resist applied load and that, in some instances, the various structures are carrying load in different parts of the skull.

### The relationship between morphology and performance

Comparisons between linear measurements of shape and performance showed that, within the cranium, tooth row length (TRL) was the best predictive variable for strain during bite and shake loading whilst dorsal cranial length (DCL) was the best predictive variable for pull loading. Mandible length (ML) was found to be the best predictor for strain levels during bite, shake and pull loading in the mandible (Table 6). In all cases larger values corresponded with higher levels of strain.

**Table 6:** Best linear morphometric predictor variables for each load case.

Cranium				Mandible			
Load Case	Best Predictor	n.o of factors	% explained	Load Case	Best Predictor	n.o of factors	% explained
Bite	TRL	1	90.24	Bite	ML	1	69.96
Shake	TRL	1	92.11	Shake	ML	1	81.99
Pull	DCL	1	83.28	Pull	ML	1	65.68

Partial least squares regression identified that the cranial shape was closely related to the response of models to bite and shake loading (Table 7). The relationships were however less strong in the mandible (Percentage variation explained for cumulative Y = 40.58 and 26.22 for the cranium and mandible respectively). In both the cranium and the mandible shake loading had a stronger relationship to shape than biting, and both of these were stronger than pulling (Table 7). Variable Importance Values of 1.4130 and 1.4142 for the PC1 in the cranium and mandible respectively, and 0.0582 and 0.0068 for PC2 in the cranium and mandible respectively indicate that PC1 values were far more important in these relationships.

**Table 7:** Statistical comparison (PLS) between morphology (PC1 and PC2) and Performance (Bite, Shake and Pull load cases).

Cranium	n.o of factors	% explained	Mandible	n.o of factors	% explained
Bite	1	41.02	Bite	1	27.87
Shake	1	52.19	Shake	1	38.46
Pull	1	28.53	Pull	1	12.34

### The relationship between performance and ecology

No relationships were observed between the presence of large prey in the diet of species and the structural performance of either the cranium or the mandible in any type of loading (Table 8). Relationships were observed between the presence of hard prey items within the diet of the species and the structural performance of cranial and mandible models; species that fed on hard prey items performed with less strain in bite loading than other species.

**Table 8:** Univariate Statistical comparison (ANOVA) between ecological class and performance in each load case.

Cranium			Mandible		
Hard		Prob>F	Hard		Prob>F
	Bite	**0.0106**		Bite	**0.0064**
	Shake	0.7469		Shake	0.1207
	Pull	0.9016		Pull	0.1083
**Large**		**Prob>F**	**Large**		**Prob>F**
	Bite	0.1788		Bite	0.1636
	Shake	0.0887		Shake	0.0886
	Pull	0.2995		Pull	0.2660

## Discussion

We aimed to investigate the relationships between the cranial morphology, biomechanical performance (VM strain under biting, pulling and shaking loads) and feeding ecology of varanoid lizards. There were strong relationships between shape and performance; crania and mandibles with larger widths and heights (high PC1 values) performed better at biting and shaking. Further, there was some evidence for relationships between performance and diet; species that performed well in bite loading were also found to have hard prey within their diet. However, the relationships between morphology and performance were not as evident as that reported in previous studies [21,75], and did not match predictions from mechanical first principles, or those identified by FEA in other taxa [21].

### Ecomorphology

Species that feed on large prey clustered together in the bottom right of both the cranial and mandible morphospace (gracile with long rostrum). Species that do not feed on hard prey items clustered in the centre top of cranial morphospace exhibiting low cranial height. There was a clear relationship between prey size and mandible shape, with macrophagous species having lower mandible PC1 and PC2 values. Phylogenetic patterns were also present, for example dwarf monitor (subgenus *Odatria*) mandibles clustered together in the morphospace.

The most extreme form of feeding upon hard prey is durophagy, *Varanus exanthematicus* and *L*. *borneensis* are both durophagous, feeding on hard prey items (molluscs and crabs respectively) [59,76]. *Heloderma horridum*, although not distinctively durophagous, does also feed on harder prey items such as eggs. These three species have the highest PC1 scores for both crania and mandibles (Figs 6 & 7), indicating that, as expected, the shortest and most robust skulls are those suited for durophagy. Note, however, that phylogeny is also important here; durophagy may be a plesiomorphic trait of the varanoid lizards as *Varanus exanthematicus* and *L*. *borneensis* are basal species.

### Loading type

In simulations of biting, the models consistently showed higher strain levels in the mandible than the cranium. Biomechanical performance during biting may thus be limited primarily by mandibular morphology, as has been suggested in previous analyses [77]. This may also be a result of differing evolutionary forces influencing the two structures; as the cranium houses the brain and capsules, its morphology is a result of multivariate selection, whereas the mandible is principally involved in feeding [78]. The high strain levels observed in the brain case and in between the orbits during shake loading indicate that these areas may be important in this behaviour (Fig 9). This raises the possibility of a trade-off between eye size (sensory function) and strength during shaking (structural function), consistent with a previous study on theropod dinosaurs [79]. The two sub-adult specimens included within the analysis (*V*. *komodoensis* and *V*. *gouldii)* exhibited higher magnitudes of strain than most other specimens. Eyes are proportionately larger in juveniles, leaving less space for bone in the interorbital region, and that region exhibits high level of strain during biting and shaking. Thus fine scale structural characteristics such as allometric differences between juveniles and adults may be of particular importance to trade-offs in cranial structural performance and neurocranial anatomy.

We examined correlations between morphology and performance using two measures of morphology: linear (euclidean distances), and multivariate (geometric morphometrics).

### Linear morphometrics vs performance

Walmsley et al. (2013) found that relationships between linear morphometrics and performance match well with predictions from mechanical first principles in a comparative analysis of crocodile mandibles. However, relationships between linear measures of shape and performance results from FEA in our study were inconsistent with our predictions: the length of the tooth row and mandible correlated better with skull strength (Table 6) than the measures we hypothesised a priori (i.e. rostral/mandibular height for biting, rostral/mandibular width for shaking, and the combination of cranial/mandibular height and width for pull back loads).

### Multivariate morphometrics and performance

In the principle components analysis of skull shape, PC1 values correlated negatively with skull length and positively with skull width, so that species with high PC1 values had shorter, wider skulls with taller snouts (Figs 3 and 4). *A priori*, these would be expected to be stronger under biting and especially shaking loads, and the results agree; two blocked partial least squares regression showed that crania and mandibles with high PC1 values performed better than others, particularly in bite and shake loading (Table 7). Species with lower PC2 values have taller skulls post-orbitally, which is also expected to increase skull strength, but this had a smaller influence on strain levels as indicated by comparable lower variable importance values in the 2-block partial least squares analysis. The fact that there were higher explanation values for biting and shaking load cases may mean that performance in these behaviours is more important than performance in pulling behaviours in driving patterns of morphological variation in the varanoid skull.

### Overall

The apparent mismatch between the linear measurements that were predicted to influence skull performance, and those that actually do, may relate to the space frame structure of the varanoid lizard cranial system [36]. Compared with the shell constructions, it seems that the performance of varanoid skulls is difficult to predict from simple measurements chosen with reference to basic beam theory. However, more complex multivariate measures of skull shape do show correlations with performance that make sense in terms of fundamental mechanical principles. If this is the case, then the landmarks used in the multivariate measures must somewhere incorporate aspects of shape that do influence performance. The higher correlation between morphology and performance in the cranium than in the mandible may indicate that the cranium is under a higher selective pressure for strength; intuitively this is difficult to understand as cranial morphology is expected to represent compromise between various functional demands (feeding, protecting the brain, olfaction etc.) whilst the mandible primarily functions in feeding [78].

*V*. *komodoensis* exhibited high levels of strain to bite, shake and pull loading. This result is may be a consequence of our model being constructed from a sub-adult specimen (a size that cannot exploit large prey) that has no ecological need for a robust skull. Alternatively it is also possible that the use of toxic effects (whether produced by bacteria or venom glands) to incapacitate prey may free the Komodo dragon from the need to have a highly robust skull [45,80,81]. Clearly further research that measures cranial performance across ontogeny may detail allometric changes in performance consistent with dietary transitions from small prey in hatchlings to large ungulates (e.g. water buffalo) in adults [82].

### Do macropredaceous varanoids have longer, weaker skulls?

Species that fed on large prey items compared to their size possess unique morphological characteristics such as elongate crania and mandibles. Predatory mammals and crocodiles that feed on large prey exhibit the opposite trend with shorter, more robust cranial structures [24,75]. This may be due to the influence of other performance considerations such as the need for a long mandible to better tear flesh during pull back prey processing. At least some of the varanid species that feed on large prey use pull back behaviours to process their food. In *V*. *komodoensis* the tooth row acts as a saw blade which cuts food as the teeth are drawn back [15]. Logically, an elongate mandible can carry more teeth and thus increases the efficiency of prey processing. Many of these species also feed on carrion which may mean that the skull does not need to handle the same level of extrinsic forces that can occur in high levels during prey capture [59]. Alternatively the elongate skulls could prove useful in allowing the predator to better access hard to reach parts of the carcass.

### Methodological limitations and agenda for future research

The complexity of varanoid morphology, behaviour and ecology represented a significant challenge for this study. The relationships identified between morphology and performance were not as strong as those identified in other taxonomic groups [21,24,75]. Either there is only a weak link between morphology, performance and ecology within the group (with behaviour acting as a wild card), or there are limitations on the approach that we used here.

#### Sample size

Many previous FEA studies have characterised the biomechanics of a single specimen or qualitatively compared between a small number of species [25,26,27,37,45,48]. The complex morphological differences that exist between the varanoid specimens mean that a higher number of individuals are more suitable to tease apart the relationship between form and function. Additionally, a limited sample size prevents us from characterising the effects of intraspecific variation such as allometry or sexual dimorphism. Ontogenetic changes in skull shape have been shown to be large within some varanid species [83]. Sample size thus represents a substantial hurdle for the area of ecological biomechanics given that finite element models take considerable time to produce.

#### Structural complexity

The space-frame nature of the varanoid cranial system represented another significant challenge for this study. The strength of a structure is dependent on many characteristics of the shape such as the thickness of bones or their orientation. Gross morphology was found to have a large influence (PC 1 correlated with some measures of performance). However, subtler morphological characteristics, such as thicknesses of particular struts, that were not measured, are likely to also be important in dictating strength. It is logical that finer morphological characteristics also play a larger role in dictating strength in space frame structures compared to shell structures. This is because space-frame structures rely on fine scale and localised morphological features more than shell structures that distribute load across a wide surface. Sutures were not included in the analysis; these features have been found to have considerable influence on FEA results in previous studies [40,49,84,85,86,87]. Due to a lack of information on the nature and extent of cranial kinesis (movement between the bones of the cranium) in varanoids it was excluded in FEA models and it is likely that this has also influenced the results to some degree. The low percentage of total variance explained by the PC analyses could also be due to the occurrence of cranial kinesis which would alter the relative position of landmarks between specimens.

#### Muscle anatomy

Because detailed data on muscle activation and anatomy was not available for the majority of these species we were forced to assume that all species possess identical muscle attachment sites and that each muscle is used at the same time in biting. This may not be a realistic assumption; it would be useful for future studies to address the function of specific muscles and the role of muscle activation patterns.

#### Loading conditions

Recent sensitivity studies have identified that biological assumptions may have more influence on FEA results then those about material properties or scaling [57]. As no kinematic data was collected for this study it is possible that the forces applied were not exactly biologically equivalent. Future studies should aim to collect data on the exact loading conditions encountered in each species through observation of either wild or captive animals.

#### Ecological data

The performance and shape data used in this study are fine grained whereas the ecological data are quite course, with only qualitative categories being drawn. It is possible (even likely) that clear relationships between skull shape, biomechanics and ecology do exists but that fine scale ecological data are required to demonstrate them statistically. However this hypothesis cannot be tested without improving the available ecological data, and so quantitative studies of varanid ecology are a priority for investigating form-function relationships in this taxon.

#### Performance metrics

Within this study we have only examined strength (limited to three load cases) as a performance measure. As a complex integrated system other factors such as the ability of the skull to protect vital organs, the ability of the cranial system to produce bite forces and for certain sensory organs to function, are likely to also influence the shape of the skull [88].

## Conclusions

This study identifies that varanoid lizards do exhibit considerable variation in cranial and mandibular morphology that has specific influences on structural performance during biologically relevant loading. This could be an important characteristic that allows for these closely related taxa to exploit diverse ecological habits. The relationships between the morphology of varanoid lizard skulls, their structural performance and their ecology are not as would be predicted from our previous knowledge of cranial form and function. The space frame structure of the varanoid lizard skull as well as the unique behaviours that are used by these species to capture and process prey items may be the cause of this discrepancy. It is hoped that the insights gained from this study will help to guide future research on space-frame skull structures and more broadly in the area of ecological biomechanics. The results may also provide useful context for future paleobiological studies on taxa such as theropod dinosaurs that may use varanoids as extant morphological analogues.

## Supporting Information

S1 File Measurement definitions and Landmark locations. (DOCX)

S2 File Supplementary section containing R code for statistical analysis. (TXT)

S3 File Instructions on the use of the R code used to generate statistical summaries. (RTF)

S1 Table Supplementary data on muscle forces applied to models. Natural forces represent the force per truss element predicted for each of the muscle beams based on muscle cross sectional area at natural size. Adjusted forces represent the force per truss element adjusted so that the models produced the same node reaction force at the bite location in volume scaled models. Total muscle forces represent the total force applied to each model to generate the same node reaction forces at bite location.
(XLSX)
