# Supplementary material for: The Relationship between Cranial Structure, Biomechanical Performance and Ecological Diversity in Varanoid Lizards
Source: PLoS One. 2015 Jun 24;10(6):e0130625. doi: 10.1371/journal.pone.0130625 (PMC4479569; doi:10.1371/journal.pone.0130625)
Supplement: S1 File — (DOCX) [file pone.0130625.s001.docx]

**Measurement definitions and Landmark locations – Appendix S1**

Table S1: Definitions of the linear measurements used in this study.

| **Abbreviation** | **Definition of measurement** |
| --- | --- |
| DCL | Distance between the anterior tip of the pre maxilla and the medial, posterior margin of the parietal |
| BSL | Distance between the anterior tip of the pre maxilla and the posterior tip of the basioccipital |
| CW | Largest width of the cranium |
| CH | Largest height of the cranium |
| ML | Distance from the anterior tip of the mandible to the posterior tip of the articular |
| MW | Largest width of the mandible |
| MH | Largest height of the mandible |
| RH | Height of the cranium at the posterior margin of the maxilla |
| RW | Width of the cranium at the posterior margin of the maxilla |
| MHD | Height of the mandible at the posterior margin of the dentary |
| MWD | Width of the mandible at the posterior margin of the dentary |
| TRL | Linear distance from the tip of the premaxilla to the posterior of the maxilla |

Table S2: landmarks (points and curves) used in the geometric morphometric analysis of the crania.

| Points |  |
| --- | --- |
| 1 | Most anterior point on the skull |
| 2 | Left posterior medial tip of the postfrontal +post orbital (left side) |
| 3 | Right posterior medial tip of the postfrontal +post orbital (right side) |
| 4 | Most posterior point of the occipital condyle |
| 5 | Most posterior tip of the pterygoid (left side) |
| 6 | Most posterior tip of the pterygoid (right side) |
| 7 | Left lateral edge of the bottom of the epipterygoid (left side) |
| 8 | Right lateral edge of the bottom of the epipterygoid (right side) |
| 9 | Left lateral edge of the top of the epipterygoid (left side) |
| 10 | Right lateral edge of the top of the epipterygoid (right side) |
| 11 | Most medial point on the posterior dorsal edge of the parietal |
| 12 | Most posterior tip of the bottom left lobe of the basisphenoid |
| 13 | Most posterior tip of the bottom right lobe of the basisphenoid |
| 14 | Most anterior tip of the bottom left lobe of the basisphenoid |
| 15 | Most anterior tip of the bottom right lobe of the basisphenoid |
| 16 | Most medial point on the posterior supraoccipital |
| 17 | Most anterior point on the left top bulge of the basisphenoid |
| 18 | Most anterior point on the right top bulge of the basisphenoid |
| 19 | Left lateral posterior tip of the palatine (left side) |
| 20 | Right lateral posterior tip of the palatine (right side) |
| 21 | Most anterior intercept of the frontal and prefrontal (left) |
| 22 | Most anterior intercept of the frontal and prefrontal (right) |
| 23 | Left lateral tip of the quadrate (left) |
| 24 | Right lateral tip of the quadrate (right) |
|  |  |
| curves |  |
| 1 | Left lateral, ventral edge of the maxilla (left side) |
| 2 | Right lateral, ventral edge of the maxilla (right side) |
| 3 | Ridge in slope on the dorsal surface of the parietal (left side) |
| 4 | Ridge on the dorsal surface of the parietal (right side) |
| 5 | Dorsal posterior edge of the parietal (left) |
| 6 | Dorsal posterior edge of the parietal (right) |
| 7 | Lateral surface of the pterygoid (left) |
| 8 | Lateral surface of the pterygoid (right) |
| 9 | Dorsal surface of the maxilla (left) |
| 10 | Dorsal surface of the maxilla (right) |
| 11 | Medial line on the dorsal edge of the premaxilla |

Table S3: landmarks (points and curves) used in the geometric morphometric analysis of the mandibles.

| Points |  |
| --- | --- |
| 1 | Most anterior point on the dentary (left side) |
| 2 | Most anterior point on the dentary (right side) |
| 3 | Most dorsal point on the coronoid process (left side) |
| 4 | Most dorsal point on the coronoid process (right side) |
| 5 | Most posterior point on the articular (left side) |
| 6 | Most posterior point on the articular (right side) |
| 7 | Dorsal meeting of the dentary and the cornoid (left side) |
| 8 | Dorsal meeting of the dentary and the cornoid (right side) |
| 9 | Ventral meeting of the dentary and the angular (left side) |
| 10 | Ventral meeting of the dentary and the angular (right side) |
| 11 | Medial dorsal point on the articular (jaw hinge) (left side) |
| 12 | Medial dorsal point on the articular (jaw hinge) (right side) |
| 13 | Lateral intercept of the dentary, coronoid and surangular (left side) |
| 14 | Lateral intercept of the dentary, coronoid and surangular (right side) |
|  |  |
| curves |  |
| 1 | Dorsal left lateral edge of the dentary (left side), from the first to the last tooth position |
| 2 | Dorsal right lateral edge of the dentary (right side), from the first to the last tooth position |
| 3 | Ventral, medial edge of the dentary (right side), from directly under the second tooth position to under the last tooth position |
| 4 | Ventral, medial edge of the dentary (left side), from directly under the second tooth position to under the last tooth position |
| 5 | Medial edge of the coronoid between the dentary and most dorsal point (left side) |
| 6 | Medial edge of the coronoid between the dentary and most dorsal point (right side) |
| 7 | Medial edge of the coronoid process from the most dorsal point to the surangular (left) |
| 8 | Medial edge of the coronoid process from the most dorsal point to the surangular (right) |
| 9 | Medial edge of the dentary between the intamandibular joint and the last tooth position (left side) |
| 10 | Medial edge of the dentary between the intamandibular joint and the last tooth position (right side) |
| 11 | Edge of the dentary ventral to the tooth row (left side) |
| 12 | Edge of the dentary ventral to the tooth row (right side) |
| 13 | Ventral lateral edge of the coronoid (left side) |
| 14 | Ventral lateral edge of the coronoid (right side) |
| 15 | Medial edge of the articular between the jaw joint and the most posterior point (left side) |
| 16 | Medial edge of the articular between the jaw joint and the most posterior point (right side) |
| 17 | Lateral edge of the articular between the jaw joint and the most posterior point (left side) |
| 18 | Lateral edge of the articular between the jaw joint and the most posterior point (right side) |
| 19 | Posterior margin of the mandible between the dentary and the posterior tip of the articular (left side) |
| 20 | Posterior margin of the mandible between the dentary and the posterior tip of the articular (right side) |
| 21 | Lateral edge of the surangual between the coronoid and the jaw joint (left side) |
| 22 | Lateral edge of the surangual between the coronoid and the jaw joint (right side) |
