# Supplementary material for: The Relationship between Cranial Structure, Biomechanical Performance and Ecological Diversity in Varanoid Lizards
Source: PLoS One. 2015 Jun 24;10(6):e0130625. doi: 10.1371/journal.pone.0130625 (PMC4479569; doi:10.1371/journal.pone.0130625)
Supplement: S3 File — (RTF) [file pone.0130625.s003.rtf]

Instructions for getsummaries

getsummaries

Getsummaries produces excel files containing the following statistical information for multiple files:
(i) Min, Max, Mean, standard deviation
(ii) Percentiles: 0.25,0.5,0.75,0.9,0.95,0.98,0.99,0.999

It contains the following functions:
columninput
croporcompletedata
filelayout
getfilenames
getmultistrstrdata
getnofiles
getpermultidata
getreducedmultidata
gettoppercentsmultidata
getwritefile
keeppercentages
keeptoppercent
makematricx
removelowervalues
yesno

summaries

How to use:
(1) Open R
(2) Select file and change dir... to change the working directory which contains your data files.
(3) Select file, open script and open getsummaries.
(4) Select edit, run all or press alt+e+n (This runs the script so you can use it.)
(5) Type summaries() into the many R console and hit enter. This starts the program.

(6) After asking for the number of files you wish to compare, the program will ask you if you wish to use the existing data retention levels of 99%, 97% and 95%.
    The program computes the statistical summeries on the complete data set and also three other data sets which are formed from removing some of the top data points. For example, the 99%
    retention level removes the top 1% of data. These three values can be changed by pressing 2. 1 keeps them.
(7)  of your files, you need only enter the file names WITHOUT the .txt extension. R is not 
case-sensitive, but any other mistakes and it will crash the program.
    IF you didn't change the working directory you need to enter the full address starting from "C:/". In R, where Windows using a "\", R uses a "/".

(8) The program now asks you for a save to file name. Omit the .xls extension.
    The excel file will be saved in the working directory, unless the whole address is given, similar to (7).
(9) Are the text files in the default layout? The default layout consists of 18 skipped lines and then 3 columns, one non-numerical and two numerical. The column of interest is the third. If this is the setup of your text file than enter 1, otherwise enter 2 and follow the screen prompts.
(10) Do you wish to remove data below a certain value? Perhaps there are many data points around 0, and they are making it difficult to see what is happening in the rest of the dataset. Perhaps data below a certain value is extremely uninteresting, this is a way you can remove it.
(11) Do you wish to analyse a proportion of the highest data points? This allows you to focus in on just the top Y% of data.
(12) When there is a red greater than sign on the R console, the program has finished. The excel file will be made and saved as in part (7).
